# Ensuring Accuracy and Equity in Vaccination Information From ChatGPT and CDC: Mixed-Methods Cross-Language Evaluation

**DOI:** 10.2196/60939

**Published:** 2024-10-30

**Authors:** Saubhagya Joshi, Eunbin Ha, Andee Amaya, Melissa Mendoza, Yonaira Rivera, Vivek K Singh

**Affiliations:** 1 School of Communication & Information Rutgers University New Brunswick, NJ United States

**Keywords:** vaccination, health equity, multilingualism, language equity, health literacy, online health information, conversational agents, artificial intelligence, large language models, health information, public health

## Abstract

**Background:**

In the digital age, large language models (LLMs) like ChatGPT have emerged as important sources of health care information. Their interactive capabilities offer promise for enhancing health access, particularly for groups facing traditional barriers such as insurance and language constraints. Despite their growing public health use, with millions of medical queries processed weekly, the quality of LLM-provided information remains inconsistent. Previous studies have predominantly assessed ChatGPT’s English responses, overlooking the needs of non–English speakers in the United States. This study addresses this gap by evaluating the quality and linguistic parity of vaccination information from ChatGPT and the Centers for Disease Control and Prevention (CDC), emphasizing health equity.

**Objective:**

This study aims to assess the quality and language equity of vaccination information provided by ChatGPT and the CDC in English and Spanish. It highlights the critical need for cross-language evaluation to ensure equitable health information access for all linguistic groups.

**Methods:**

We conducted a comparative analysis of ChatGPT’s and CDC’s responses to frequently asked vaccination-related questions in both languages. The evaluation encompassed quantitative and qualitative assessments of accuracy, readability, and understandability. Accuracy was gauged by the perceived level of misinformation; readability, by the Flesch-Kincaid grade level and readability score; and understandability, by items from the National Institutes of Health’s Patient Education Materials Assessment Tool (PEMAT) instrument.

**Results:**

The study found that both ChatGPT and CDC provided mostly accurate and understandable (eg, scores over 95 out of 100) responses. However, Flesch-Kincaid grade levels often exceeded the American Medical Association’s recommended levels, particularly in English (eg, average grade level in English for ChatGPT=12.84, Spanish=7.93, recommended=6). CDC responses outperformed ChatGPT in readability across both languages. Notably, some Spanish responses appeared to be direct translations from English, leading to unnatural phrasing. The findings underscore the potential and challenges of using ChatGPT for health care access.

**Conclusions:**

ChatGPT holds potential as a health information resource but requires improvements in readability and linguistic equity to be truly effective for diverse populations. Crucially, the default user experience with ChatGPT, typically encountered by those without advanced language and prompting skills, can significantly shape health perceptions. This is vital from a public health standpoint, as the majority of users will interact with LLMs in their most accessible form. Ensuring that default responses are accurate, understandable, and equitable is imperative for fostering informed health decisions across diverse communities.

## Introduction

There is a growing recognition of the role of information as a crucial determinant of health [1]. Globally, Google witnesses more than 100 million daily health-related searches. Similarly, Open AI’s ChatGPT experiences over a billion monthly visits and is increasingly used in medical contexts [2]. A survey reports more than 80% of US respondents had used a chatbot in 2023, and another suggests that despite prohibitions on medical use by vendors, millions of medical queries are submitted on a weekly basis by users of OpenAI alone [3,4]. Such publicly available large language models (LLMs) such as ChatGPT can be a promising source of health care information. Individuals may readily derive benefits from straightforward queries and interactive dialogue when seeking medical advice or making health-related decisions. However, evaluations on the quality of LLM responses still show conflicting results. Recent studies reveal that human experts perceived ChatGPT’s responses to be accurate, relevant, easy to read, and comprehensive [5-7]. Despite the potential usability of LLMs, scholars pose concerns about the use of ChatGPT and other LLMs for professionals’ medical advice [8,9]. Empirical evidence still exists for plausible-sounding yet inaccurate or fraudulent outcomes, as well as limited readability, semantic repetition, or coherence loss in lengthy passages [8,10-13].

Notably, there is a critical need to examine how LLMs respond to controversial topics such as vaccination. Communication and media environments can be regarded as determinants of hesitant vaccine attitudes [14]. Given the emergence of LLMs such as ChatGPT as channels of health information, their responses may shape users’ perceptions of the vaccine and health care decision-making. Recent work suggests that ChatGPT exhibits a notably precise, clear, easy-to-understand, and unbiased tone in its delivery of vaccination [9,15,16]. Yet, most research has solely focused on the quality of ChatGPT responses in English, limiting considerations of linguistic equity.

Scholars have paid relatively little attention to LLMs' multilingual capabilities. Given the potential impact of language barriers and linguistic inequities in health care [17-20], the evaluation of their multilingual outcomes should become an integral part of ensuring health equity. Indeed, there are remarkable disparities in vaccination coverage and attitudes by racial and language groups in the United States. For example, Latino and Black populations were more hesitant to COVID-19 vaccines than White populations [21]. Latino parents have also shown a high rate of COVID-19 vaccine resistance and uncertainty [22,23]. Furthermore, Latino adults have reported lower human papillomavirus vaccination rates (41%) than White and Black populations (50% and 46%, respectively) [24].

Given the need to increase information access and equity surrounding vaccines in non-English languages for those with low English preference, we argue that we should pay attention to the different linguistic features of ChatGPT responses, particularly as it relates to Spanish, the most spoken non-English language in the United States [25]. It is necessary to comprehensively evaluate LLMs’ multilingual outcomes with consideration to both response quality and equity. However, this area is severely understudied. A table of related works has been presented in Table 1. As summarized in the table, the unique contribution from this study is the mixed methods approach to compare ChatGPT responses across multiple languages and multiple dimensions using a validated instrument such as the National Institutes of Health’s Patient Education Materials Assessment Tool (PEMAT) to measure understandability, level of misinformation to measure accuracy and Flesch-Kincaid readability and grade Level to measure readability. Other qualitative studies have been conducted using PEMAT and Flesch-Kincaid grade levels [6,8], but only for English language. There are very few studies that compare across multiple languages using both quantitative and qualitative evaluation. One notable exception is the study by Jin et al [26], but it does not use validated instruments. Another exception is our own previous work, which found the disparity in vaccine hesitancy-related responses across different languages [27]. Though we found that vaccine-hesitancy was the most in English responses and the least in Spanish responses, the study was limited to comparing single-word quantitative responses with vaccination survey questions in English, Spanish, and French. To ensure qualified and equitable health information in multilingual LLMs, we need more research that examines the cross-language health content across diverse dimensions. These works are tabulated in Table 1 where we see no other work using mixed methods to compare responses across languages using validated scales.

**Table 1:** Comparison of related works that study large language model responses in health context.

Study	Dimensions				Coders		Multilingual		Method
	Accuracy	Understandability	Readability						
Johnson et al [6]	—^a^	—^a^	FK^b^	5 human experts		No		Qualitative comparison between ChatGPT responses and the National Cancer Institute’s frequently asked questions (FAQs)	
Pan et al [8]	Level of misinformation	PEMAT^c^	FK^b^	2 human experts		No		Cross-sectional study of quality of info. across 4 chatbots	
Jin et al [26]	Auto + human	—^a^	—^a^	LLM + human		Yes		quantitative as well as qualitative evaluation across multiple languages	
This study	Level of misinformation	PEMAT^c^	FK^b^	3 coders		Yes		Mixed methods to compare ChatGPT responses across multiple languages and dimensions	

^a^—: not applicable.

^b^FK: Flesch-Kincaid.

^c^PEMAT: Patient Education Materials Assessment Tool.

The current study aims to expand our previous work by exploring whether popular LLMs, such as ChatGPT, provide reliable health information in multiple languages. We specifically aim to compare responses to childhood vaccination–related frequently asked questions from the Centers for Disease Control and Prevention (CDC) and ChatGPT across accuracy, understandability, and readability dimensions in both, English and Spanish using both, quantitative and qualitative approaches. By doing so, we hope to identify and address systemic inequities that may exist in LLMs, so as to better promote equity and inclusion in the development of technologies that impact access to quality health information and communication.

## Methods

### Data Collection

**Figure 1:**
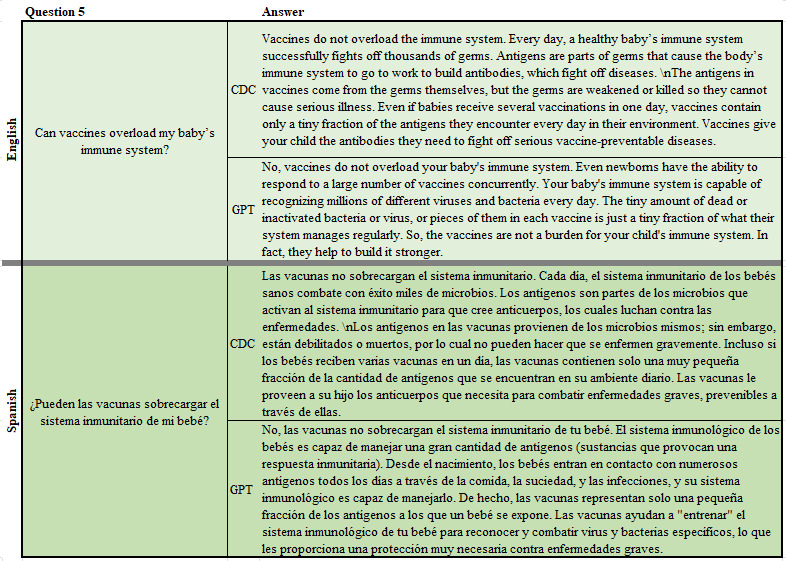
A sample question from the frequently asked questions and sample answers as obtained from Centers of Disease Control and Prevention and ChatGPT.

In order to examine the variation in ChatGPT response, we used a set of 16 frequently asked questions (FAQs) on the CDC website in both English and Spanish [28,29]. Chat completion API from OpenAI was used to interface with the ChatGPT-4 engine to obtain textual responses to each of the questions [30]. These questions yielded short free-form responses. Figure 1 shows an example of a single response. This process was repeated 3 times to avoid extreme single responses impacting the results dramatically. Overall, we had a total of 96 responses from ChatGPT (3 responses for each of the 16 questions in both languages) and 32 responses from CDC’s FAQs (16 responses each in both languages, 128 responses in total).

### Quantitative Analysis

Responses were evaluated on 3 dimensions, namely, accuracy, understandability, and readability. These dimensions were identified based on their importance in the literature and the potential impact that they can have in contentious health contexts [5-11,31-34].

Accuracy was assessed using a 3-point item to measure the level of misinformation in responses (1=no misinformation, 2=some misinformation, and 3=high misinformation). Multimedia Appendix 1 [35-42] includes our reasoning for using a 3-point scale. Understandability was assessed using the National Library of Medicine’s Health Education Materials Assessment Tool [31], which consists of 8 yes or no items adapted from the Patient Education Materials Assessment Tool’s understandability domain [32]. A final score was derived as an average of the 8 items and represented as a percentage, with higher scores meaning higher understandability. This instrument is available in English and Spanish (Multimedia Appendix 1 [35-42]). Two coders [AA] and [MM] independently scored blinded responses for accuracy and understandability. Coders were bilingual, bicultural students trained by a bilingual, bicultural team member who is an expert in qualitative data analysis in health communication and public health. Interrater reliability was high for both domains (98% agreement in accuracy, 𝜅=–0.01; 99% agreement in understandability, 𝜅=0.86) [43,44]. Readability was assessed using Flesch-Kincaid readability scores for English and Flesch-Huerta index for Spanish [33,34] where scores between 0 and 100 are scaled to grade levels from fifth grade (90-100) to professional (0-10). Data was extracted and summarized using Python and transformed into spreadsheets; basic statistical tests were conducted using Microsoft Excel (version 2312).

### Qualitative Analysis

We also conducted a qualitative analysis of the responses, with the goal of providing additional context regarding any nuances within and between languages that would otherwise not be captured (eg, typographical errors, sentence structure, and word choice) [45]. Coders were provided instructions for each of the domains assessed, then provided with space to take notes of any important nuances they saw in any of the item’s responses as related to overall tone (sentence structure, word choices, particularly in Spanish, or spelling nuances) to discuss as a team. This was accomplished using a Qualtrics form, where coders were instructed to enter any observations regarding the similarities and differences within and between languages for each set of responses.

The coders then proceeded to discuss all items and notes with the lead faculty member [YR] and achieve consensus on findings to ensure dependability and reliability in assessments and identify similarities and differences in responses between languages and achieve consensus to ensure dependability and reliability in assessments [46,47].

## Results

### Quantitative Analysis

Table 2 shows average word, sentence, and syllable counts. On average, Spanish used more words, sentences, and syllables per response. ChatGPT responses were generally more verbose than CDC responses. In addition, ChatGPT sentence count ranges were more variable than those of CDC responses for both English (ChatGPT: 1-22 and CDC: 2-7) and Spanish (ChatGPT: 2-24 and CDC: 2-8).

The results in terms of accuracy, understandability, and readability are summarized in Table 3.

**Table 2:** Mean (range) of verbosity measures.

Measures	English		Spanish			Total	
	CDC^a^, mean (range)	ChatGPT, mean (range)	CDC^a^, mean (range)	ChatGPT, mean (range)	CDC^a^, mean (range)		ChatGPT, mean (range)
Sentence count	4.06 (2-7)	7 (1-22)	4.38 (2-8)	7.15 (2-24)	4.22 (2-8)		7.07 as (1-24)
Syllable count	116.75 (53-241)	172.79 (63-515)	189.94 (82-371)	270.5 (100-672)	153.34 (53-371)		221.65 (63-672)

^a^CDC: Centers for Disease control and Prevention.

**Table 3:** Mean (range) of different attributes in English, Spanish, and total for CDC and ChatGPT.

Attributes	English			Spanish			Total	
	CDC^a^, mean (range)	ChatGPT, mean (range)	CDC^a^, mean (range)		ChatGPT, mean (range)	CDC^a^, mean (range)		ChatGPT, mean (range)
Number of sessions with ChatGPT	16	16 × 3	16		16 × 3	2 × 16		2 × 16 × 3
Accuracy	1 (1-1)	1.02 (1-2)	1 (1-1)		1.01 (1-2)	1 (1-1)		1.02 (1-2)
Understandability	95.65 (85.7-100)	95.87 (57.1-100)	98.83 (75-100)		95.7 (71.43-100)	97.24 (75-100)		95.79 (57.14-100)
Readability score	48.1 (26.61-65.93)	42.52 (22.36-62.1)	74.92 (53.65-89.02)		69.63 (54.56-80.72)	61.51 (26.61-89.02)		56.08 (22.36-80.72)
Grade level	12.13 (8.5-16)	12.84 (8.5-16)	7.19 (6-11)		7.93 (6-11)	9.66 (6-16)		10.39 (6-16)

^a^CDC: Centers for Disease Control and Prevention.

#### Accuracy

We found that all responses had high accuracy. CDC responses in both languages had no misinformation, while only 3 responses were coded as having some misinformation by 1 coder each (due to nuanced responses lacking clarifying context). None of the responses were rated as having high misinformation.

#### Understandability

Responses also rated high in understandability (Table 3). There were no significant differences in understandability between CDC and ChatGPT responses within or between languages (Table S5 in Multimedia Appendix 1 [35-42]), suggesting ChatGPT responses were in alignment with CDC messaging.

#### Readability

There was significant variation in readability scores between the responses within languages and between CDC and ChatGPT (Table 3). On average, ChatGPT responses had lower readability scores than the CDC responses, regardless of language (56.08 vs 61.51; t_23_=2.32; *P*=.03). Meanwhile, when comparing responses by language, English responses had lower average readability scores than Spanish responses for both CDC and ChatGPT (CDC: t_29_=48.10 vs 74.92; t_29_=–7.87; *P*<.001; ChatGPT: 42.52 vs 69.63; t_24_=–13.03; *P*<.001; Tables 3 and 4). When comparing grade levels, English responses for both CDC and ChatGPT were significantly higher than those in Spanish (CDC: 12th grade English vs 7th grade Spanish, *P*<.001, (df)=26; ChatGPT: 13th grade English vs 8th grade Spanish, *P*<.001, (df)=26). Given the American Medical Association’s recommendation that patient materials be written at the sixth-grade level [48], we assessed the odds of each platform in satisfying this requirement. Overall, CDC responses were 13.57 times more likely to satisfy the sixth-grade level than ChatGPT responses (Χ^2^_1_=5.6, *P*=.02; Fisher Exact *P*=.01). This was similar among Spanish language responses (CDC 15.64 times higher than ChatGPT; Χ^2^_1_=5.86, *P*=.02; Fisher Exact *P*=.01). We did not observe any significant differences between CDC and ChatGPT English responses (details on post hoc pairwise across different groups are available in Multimedia Appendix 1 [35-42]). In order to verify the effect of metric across different groups, post hoc 2-tailed pairwise *t* tests at 95% significance were conducted as shown in Table 4.

**Table 4:** Significance of difference between groups using t tests.

Attributes	English and Spanish					ChatGPT					CDC^a^			
	CDC^a^	ChatGPT	*t* test (*df*)	*P* value	English		Spanish	*t* test (*df*)	*P* value	English		Spanish	*t* test (*df*)	*P* value
Readability score	61.51	56.08	2.32 (23)	.03	42.52		69.63	–13.03 (24)	<.001	48.10		74.92	–7.87 (29)	<.001
Grade level	9.66	10.39	–1.96 (26)	.06	12.84		7.93	16.19 (21)	<.001	12.13		7.19	8.71 (21)	<.001

^a^CDC: Centers for Disease Control and Prevention.

### Qualitative Analysis

When qualitatively comparing responses in both languages, several differences were observed that provide additional context otherwise missed. For example, ChatGPT would oftentimes respond in list format, making it somewhat easier to read responses comparing risks and benefits, side effects, or other reasons to vaccinate. ChatGPT would also provide additional information and examples to questions. When specifically looking at Spanish responses, we observed some Spanish text using English words in quotations (eg, “herd immunity” and “fake”). We also noticed that, despite better readability scores than English, some Spanish responses would use less colloquial words (eg, “proporcionar” instead of “proveer” or “patógeno” instead of “infección”), while others had sentence structures that resembled a word-by-word English translation (eg, “Retrasar las vacunas puede poner en riesgo a su hijo (y a otros) de contraer enfermedades que podrían haberse prevenido” rather than “Al retrasar vacunas, su hijo y otros pueden contraer enfermedades que podrían prevenirse,” which might be more commonly used by a native Spanish speaker). All responses are provided in Multimedia Appendix 2.

## Discussion

### Principal Findings

This study evaluated the quality and equity of LLM’s outcomes. Our findings show that ChatGPT provided adequate levels of accuracy and understandability to vaccine-related questions in both English and Spanish. Past results on the accuracy of ChatGPT have been mixed. While some recent work exploring ChatGPT’s responses to health-related content also suggests little to no misinformation is being shared [6,8,27], others suggest significant levels of misinformation [11]. Our results suggest that in the context of vaccine FAQs, ChatGPT provides information with high accuracy. Furthermore, ChatGPT’s easy-to-understand responses could be an accessible resource for users with limited health literacy or with limited access to health care services, thereby contributing to efforts to address health disparities and inequities. This may be particularly useful to Spanish-speaking individuals in areas where there is limited access to language-concordant health education.

However, our study also found some challenges in the quality and equity of LLM’s outcomes. First, there is a need to moderate ChatGPT’s responses, particularly in English, to adhere to recommended reading levels. The American Medical Association-recommended reading levels for health care material are at sixth grade or below. However, ChatGPT’s English responses to childhood vaccination questions often necessitated reading skills well above that of a sixth-grade level. This was also the case with CDC. Both scenarios merit attention since failure to adhere to acceptable readability standards could act as a potential barrier to health information. Ease of reading may lead to enhanced knowledge of health, thereby playing a crucial role in taking functional health literacy [49].

Second, we observed that the representations of words in ChatGPT occasionally exhibited the linguistic patterns of English in the Spanish responses. While these were not incorrectly written, some Spanish responses seem to be translated directly from English text or used less common Spanish vocabulary. There were also several instances where the Spanish response had English words in quotation marks, even though a Spanish equivalent exists. Although it may not merely translate word-for-word between English and other languages, recent evidence found that the multilingual language model, Llama-2 (Meta), is primarily dependent on English to understand the meanings of ideas across different languages [50]. While LLMs use multilingual training data, English is still the most dominant language in their training dataset [51]. Indeed, LLMs are mostly skilled in English-based tasks and are also proficient in translating from English to non-English languages. However, such verbatim translations of English could fail to capture adequate domain-specific jargon and nuances of cultural context [52] and lead to a lack of information support for those with preferences for non-English languages to obtain public health information. Therefore, English dependency in the training data of LLMs could be a potential risk to health care equity. In the future, more inclusion of more diverse data sets from other languages including minority dialects should be considered in training data.

We note that the results presented in this work focus on those obtained without any prompt engineering. For instance, carefully crafted prompt engineering could impact the readability of ChatGPT-generated responses. Our study centers on the natural querying behavior exhibited by the majority of ChatGPT users, who typically engage with the system in a conversational manner, similar to their interactions with traditional search engines such as Google. This is particularly true for vulnerable populations seeking health information, who may not be aware of or use prompt engineering techniques. While ChatGPT has 100 million active weekly users [53], there is no clear data on how many of these users use prompt engineering. However, it is reasonable to assume that a significant portion of these users, especially those from nontechnical backgrounds, with limited English proficiency, and those under medical duress, interact with ChatGPT without advanced prompting strategies. Our research illuminates the natural user experience and the inherent readability of ChatGPT’s responses, which holds significant implications for public health informatics. The differences in responses under typical user conditions are noteworthy and warrant further examination, particularly in light of multilingual users who may be at higher risk of health inequities.

The work also intersects with recent legislation and policy discussions around guardrails needed for automated artificial intelligence (AI) systems. According to the Executive Order [54], “irresponsible use [of AI] could exacerbate societal harms such as fraud, discrimination, bias, and disinformation…” LLM implementations such as ChatGPT are classified as “automated systems” that have a direct effect on decision-making for communities due to continuous data exchange, as opposed to “passive computing infrastructure” [55]. Therefore, it is imperative that proper guardrails are put in place to maintain fairness and equity of health information by continuously monitoring the metrics such as accuracy and quality of, and access to, health information produced by LLMs like ChatGPT for everybody including underserved communities. Similarly, one of the findings of the recent report [56] from President’s Council of Advisors on Science and Technology for the president states that “Without proper benchmark metrics, validation procedures, and responsible practices, AI systems can give unreliable outputs whose quality is difficult to evaluate, and which could be harmful for a scientific field and its applications.” Since there is demonstrated disparity in the grade level of ChatGPT responses in different languages, it is imperative that thorough study of its impact in health information equity is conducted. In fact, the Office of Management and Budget issued a memo recommending “Minimum Practices for Rights-Impacting AI” [57] that involves identifying and assessing “AI’s impact on equity and fairness” and mitigating “algorithmic discrimination when it is present” by December 2024 and studies like ours are important in identifying yet understudied dimensions of health equity, that is, cross-language comparison of LLM responses in the health context.

### Limitations

This paper also has some limitations. It focuses on a single set of FAQs sourced from 1 agency (CDC) on a particular topic (vaccination). The results have only been evaluated on a single LLM technology (ChatGPT) at 1 time. As ChatGPT responses can vary over iterations, we have averaged them over 3 iterations. Our focus is limited to comparing 2 languages (English and Spanish) and future studies should consider more variations in languages, questionnaires, and information systems. However, beyond the results with a specific LLM or languages, this work aims to motivate an important area of research, equity audits across languages in different languages for health-centric conversations with automated agents.

### Conclusion

This study compared ChatGPT and CDC vaccination information in English and Spanish. We found that both sources were accurate and understandable, but ChatGPT had lower readability (higher-grade level) than CDC in both languages. Furthermore, some Spanish responses often appeared to be translations of the English ones, rather than independently generated, which could hinder information access for Spanish speakers. These findings suggest that ChatGPT is a promising tool for providing health information, but it needs to improve its readability and cultural sensitivity to ensure quality and equity. We recommend further research on the impact of natural language generation systems on public health outcomes and behaviors.

## Appendix

Multimedia Appendix 1 Additional details on methodology.

Multimedia Appendix 2 Data from ChatGPT as text in a zipped file.

Multimedia Appendix 3 Coding and readability data as MS XLSX file.

## Abbreviations

AI: artificial intelligence
CDC: Centers for Disease Control and Prevention
FAQ: frequently asked question
LLM: large language model
PEMAT: Patient Education Materials Assessment Tool

## Data Availability

Data have been made public upon acceptance. Data from ChatGPT are available as text in the zipped file in Multimedia Appendix 2. Coding and readability data are available as MS XLSX file in Multimedia Appendix 3. The survey used for coding the responses is available on Qualtrics [58].
