# Supplementary material for: Ensuring Accuracy and Equity in Vaccination Information From ChatGPT and CDC: Mixed-Methods Cross-Language Evaluation
Source: JMIR Form Res. 2024 Oct 30;8:e60939. doi: 10.2196/60939 (PMC11561424; doi:10.2196/60939)
Supplement: Multimedia Appendix 1 [file formative_v8i1e60939_app1.docx]

# SUPPLEMENTARY DETAILS ON METHODOLOGY

This is a Multimedia Appendix to a full manuscript submitted to the J Med Internet Res. Formative Research. As part of supplementary material, here we share details on the measurement of accuracy of responses, the metrics used for interrater agreement, statistical tests for understandability, the Patient Education Materials Assessment Tool adopted in this work, the process and resources used to support high degree of agreement among the coders in this work, and details on the Qualtrics form used for coding different facets (e.g., accuracy, understandability, readability) and qualitative aspects.

## Accuracy measurement

Other studies have used the industry standard 5-point Likert scale to measure the level of misinformation [1]. In our study, the accuracy level of the GPT response is based on misinformation, which is basically a binary response. However, we used the 3-point scale in order to capture any nuances in the coders’ perceptions without unnecessarily increasing the cognitive load.

## Measuring interrater agreement

We use two metrics: Cohen’s kappa statistic and interrater agreement percentage to measure interrater agreement in this work. In both the metrics scores range from 0 to 1 where higher scores represent more agreement. Interrater reliability is the extent to which two or more coders agree. In this work, the interrater reliability scores among coders were above 0.95. Though percent agreement is easier to interpret, it can also inflate the agreement level in some cases.

Cohen’s kappa statistic is a measure of interrater agreement with a correction for chance. Scores above 0.8 are generally considered as excellent agreement above chance or substantial agreement [2-3]. Kappa scores are the ratio of the number of times coders agree on observations minus the expected number of observations by chance, which is divided by total possible agreements above those expected by chance. There is a phenomenon called the Kappa paradox, which can occur in cases with skew towards one label (eg, “no misinformation” label for 99% of the entries), where a very high degree of agreement is expected between coders even by chance [4]. Even a slight degree of disagreement between coders (eg, 95% agreement) can result in a “less than chance” agreement and yield a negative kappa statistic. This occurred with our score for accuracy mainly because of the heavy bias toward no misinformation (similar to findings by [5]). In such settings Cohen’s kappa scores are not easy to interpret [2]. Hence, following prior literature and best practices, we report both Cohen’s Kappa and the percent agreement among coders, so as to provide the context of agreement. Overall, we interpret the results to suggest a substantial agreement between the coders.

## Test of significance for understandability

For understandability, the difference between languages was not significant for GPT responses (97.23 vs. 95.78, *P*=2.05, df=28) as well as for CDC (95.64 vs. 98.83, *P*=2.05, df=28). Also, the difference between CDC and GPT overall was not significant (97.23 vs. 95.78, *P*=2.05, df=29).

**Table 5.** Significance of difference between groups using t-tests

|  | **EN & ES** | | | | **GPT** | | | | **CDC** | | | |
| --- | --- | --- | --- | --- | --- | --- | --- | --- | --- | --- | --- | --- |
|  | **CDC** | **GPT** | **t-stat** | ***P*** | **EN** | **ES** | **t-stat** | ***P*** | **EN** | **ES** | **t-stat** | ***P*** |
| Understandability | 97.23 | 95.78 | 1.08 | 0.29 | 95.87 | 95.7 | .08 | .93 | 95.64 | 98.83 | -1.65 | .11 |

## Patient Education Materials Assessment Tool

**Eight items adapted from PEMAT-P**

1. The response makes its purpose completely evident.
2. The response does not include information or content that distracts from its purpose.
3. The response uses common, everyday language.
4. Medical terms are used only to familiarize audiences with the terms. When used, medical terms are defined.
5. The response uses the active voice.
6. Numbers appearing in the response are clear and easy to understand. (“N/A if no numbers”)
7. The response does not expect the user to perform calculations.
8. The response presents information in a logical sequence.

## Supporting interrater agreement and resolving conflicts

We also use PEMAT based guidelines to resolve conflicts and support interrater reliability in this work.

**PEMAT guidelines for resolving conflicts**

1. Have each rater independently rate the same two materials.
2. Identify items on which discrepancies were common.
3. Discuss each rater's rationale for the rating provided.
4. Review the PEMAT User's Guide to clarify how each item was intended to be rated.
5. Come to consensus on how the guidance in the User's Guide should be implemented in practice.
6. Repeat this process with additional materials until there is agreement on most items.

## List of frequently asked questions used in this study

Besides the above guidelines, the coders were also referred to a number of useful resources to obtain background information to help ascertain the accuracy of the answers in the presented dataset

**Vaccine safety**

1) Are vaccines safe?

2) What are the risks and benefits of vaccines?

3) Is there a link between vaccines and autism?

**Side effects**

4) What are the common side effects of vaccines?

5) Can vaccines overload my baby’s immune system?

**Schedule for vaccines**

6) Why do vaccines start so early

7) Should my child get shots if she is sick?

8) Should I delay some vaccines or follow a non-standard schedule?

9) Why can’t I delay some vaccines if I’m planning for my baby to get them all eventually?

10) If I’m breastfeeding, do I vaccinate my baby on schedule?

11) Can I wait to vaccinate my baby since he isn’t in child care?

12) Can I wait until my child goes to school to catch up on immunizations?

13) Why do adolescents need vaccines?

14) Why are multiple doses needed for each vaccine?

**Protection from diseases**

15) Do infants have natural immunity?

16) Haven’t we gotten rid of most of these diseases in this country?

## Resources made available to coders to identify misinformation

- Vaccine Types, HHS [6].
- Vaccines and Immunizations, CDC [7].
- Vaccines for Your Children, CDC [8].
- National Center for Immunization and Respiratory Disease, CDC [9].
- Vaccines and Immunization, WHO [10].

## Qualtrics form used for coding domains and qualitative assessment

1. **Accuracy**

**Does this response contain misinformation?**

- - No misinformation (no inaccurate was provided in the response)
  - Some misinformation (response lacks context, exhibits ‘cherry-picking’ of information, or is imprecise/vague)
  - High misinformation (response contains inaccurate or misleading information or flawed reasoning; uses definitive language that supports incorrect claims)

1. **Understandability**

**The response makes its purpose completely evident.**

The response has an obvious main message near the top. Think of the main message as the most important thing for the audience to remember after reading the response.

- - Disagree
  - Agree

**The response does not include information or content that distracts from its purpose.**

The response includes need-to-know information only — it skips the nice-to-know details. The idea is to focus on key information readers need in order to take action. Ask yourself whether any of the information would distract or overwhelm you if you were unfamiliar with the topic.

Longer responses are more likely to have distracting information, but there’s no set maximum length for responses to get a “yes” on this item. Keep in mind that the response does need to include all the content that’s relevant to understanding the main message.

- - Disagree
  - Agree

**The response uses common, everyday language.**

The response uses plain language that everyone can understand (e.g., “high blood pressure,” not “hypertension”). Ask yourself whether you’d understand all of the terms in the response if you didn’t know anything about the topic.

- - Disagree
  - Agree

**Medical terms are used only to familiarize audiences with the terms. When used, medical terms are defined.**

When unfamiliar terms are necessary, the response explains them in context and provides an in-text definition using easy- to-understand language. (Think: a reader just diagnosed with diabetes probably needs to learn the term “glucose.”) Ask yourself whether you’d understand all of the terms in the response if you didn’t know anything about the topic.

- - Disagree
  - Agree

**The response uses the active voice.**

The material mostly uses active voice and avoids passive voice. Ask yourself: Is the subject of the sentence **doing** the action (e.g., “experts recommend that you get a flu shot”)? If so, it’s active voice. Or is the subject **receiving** the action (e.g., “getting a flu shot is recommended by experts”)? That’s passive voice.

- - Disagree
  - Agree

**Numbers appearing in the response are clear and easy to understand. *(Select N/A if no numbers)***

Check that the material uses simple numbers — like whole numbers rather than fractions and decimals. Keep in mind that frequencies (e.g., 3 out of 10) are generally easier to understand than percentages (e.g., 30%). And make sure the material uses numerals (e.g., 3, 2nd) rather than spelled-out numbers (e.g., three, second).*

**Keep in mind that some style guides (e.g., APA style) recommend spelling out numbers under 10. Use your judgment on how to assess materials that include spelled-out numbers for this reason but would otherwise get a “yes” on all items in the assessment tool.*

**Context** for numbers — in the form of words or additional numbers, like a range — can also indicate clear use of numbers. So can **visual metaphors** (e.g., “1 ounce of cheese is about the same size as 3 dice”).

- - Disagree
  - Agree
  - N/A

**The response does not expect the user to perform calculations.**

- - Disagree
  - Agree

**The response presents information in a logical sequence.**

- - Disagree
  - Agree

**The response uses visual aids whenever they could make content more easily understood. *(Select N/A if no visuals)***

- - Disagree
  - Agree
  - N/A

**Qualitative notes re: sentence structure, word choices, spelling nuances or other issues related to overall tone**

**General comments:**

**Summary comments for English responses to this question:**

**Summary comments for Spanish responses to this question:**

**Summary comments for all responses (English and Spanish) to this question:**

References

1. Pan A, Musheyev D, Bockelman D, Loeb S, Kabarriti AE. Assessment of artificial intelligence chatbot responses to top searched queries about cancer. JAMA Oncol. 2023;9(10):1437-1440. doi:10.1001/jamaoncol.2023.2947
2. Banerjee M, Capozzoli M, McSweeney L, Sinha D. Beyond kappa: a review of interrater agreement measures. Can J Stat. 1999;27(1):3-23. [doi:10.2307/3315487](https://doi.org/10.2307/3315487)
3. Landis JR, Koch GG. The measurement of observer agreement for categorical data. Biometrics. 1977;33(1):159-174. PMID:843571
4. Bexkens R, Claessen FM, Kodde IF, Oh LS, Eygendaal D, den Bekerom MP. The kappa paradox. JSES. 2018;10(4):308. doi:10.1177/1758573218791813
5. Johnson SB, King AJ, Warner EL, Aneja S, Kann BH, Bylund CL. Using ChatGPT to evaluate cancer myths and misconceptions: artificial intelligence and cancer information. JNCI Cancer Spectr. 2023;7(2):pkad015. doi:10.1093/jncics/pkad015
6. US Department of Health and Human Services. <https://www.hhs.gov/immunization/basics/types/index.html> [accessed 2023-08–07]
7. Centers for Disease Control and Prevention (CDC). Available from: <https://www.cdc.gov/vaccines/index.html> [accessed 2023-08–07]
8. Centers for Disease Control and Prevention (CDC). Available from: <https://www.cdc.gov/vaccines/parents/index.html> [accessed 2023-08–07]
9. Centers for Disease Control and Prevention (CDC). Available from: <https://www.cdc.gov/ncird/index.html> [accessed 2023-08–07]
10. World Health Organization. Available from: <https://www.who.int/health-topics/vaccines-and-immunization> [accessed 2023-08–07]
